# Oral health-related quality of life and oral manifestations of Syrian children with congenital heart disease: a case-control study

**DOI:** 10.1186/s12903-023-03017-8

**Published:** 2023-05-23

**Authors:** Shouq Sharar Bsesa, Samir Srour, Mayssoon Dashash

**Affiliations:** 1 Department of Pediatric Dentistry, Faculty of Dentistry, Damascus University, Damascus, Syria; 2 Department of Pediatric Cardiology, The University Pediatric Hospital of Damascus, Damascus, Syria

**Keywords:** Congenital heart disease, Dental caries, Gingivitis, Index, Enamel defects, COHRQoL, Syria

## Abstract

**Background:**

There was an immense need for studies evaluating the oral health status of Syrian children with congenital heart disease (CHD) and its impact on their quality of life. No contemporary data are available. The objective of this study was to investigate oral manifestations and oral health-related quality of life (OHRQoL) of children with CHD and compare them with healthy controls aged 4–12 years.

**Methods:**

A case-control study was undertaken. A total of 200 patients with CHD and 100 healthy children belonging to the same patient’s family were included. Decayed, missed, and filled permanent teeth index (DMFT) and decayed, missed, and filled primary teeth index (dmft), Oral Hygiene Index (OHI), Papillary Marginal Gingivitis Index (PMGI), and dental abnormalities were recorded. The Arabic version of the Child Oral Health-Related Quality of Life Questionnaire (COHRQoL, 36-item) which was divided into 4 different domains (Oral Symptoms, Functional Limitations, Emotional Well-Being, Social Well-Being) were studied. Chi-square test and independent- *t-*test were used to perform statistical analysis.

**Results:**

CHD patients experienced more periodontitis, dental caries, poor oral health, and enamel defects. The dmft mean was significantly higher in CHD patients than in healthy children (5.245 vs. 2.660, *P* < 0.05). No significant difference was found between patients and controls in the DMFT Mean (*P* = 0.731). There was a significant difference between CHD patients and healthy children in the mean of the OHI (5.954 vs. 1.871, *P* < 0.05), and PMGI (1.689 vs. 1.170, *P* < 0.05). CHD patients have significantly higher enamel opacities (8% vs. 2%) and hypocalcification (10.5% vs. 2%) than controls. Also, the 4 COHRQoL domains, showed significant differences between CHD children and controls.

**Conclusions:**

Evidence about the oral health and COHRQoL of children with CHD was provided. Further preventive measures are still required to improve the health and quality of life of this vulnerable group of children.

## Introduction

CHDs are systemic diseases, that are often connected to oral diseases [1]. CHDs accounts for 28% of all major human congenital defects, with a CHD frequency of 9.1 per 1000 live birth [2].

Cardiac defects are divided into acyanotic heart defects which are more frequent, and cyanotic heart defects [3, 4].

There are several subtypes of CHDs such as ventricular septal defects (VSD) [5, 6], which are the most common, atrial septal defect (ASD) [6, 7], tetralogy of Fallot (TOF) [8, 9], pulmonary stenosis (PS) [10], and aortic stenosis (AOS) [11].

During the first few years of their lives, children with CHD are generally hospitalized for short or long periods for medical and surgical treatment [12]. For this reason, oral diseases are expected to be commonly seen in children with this disease [13]. In addition, poor oral health with early dental caries and gingivitis may be caused by additional factors such as nutrition, drug use, and family socioeconomic status [12].

Several factors such as multiple demands and stress, may distract and cause healthcare challenges to children with CHD. In these cases, oral hygiene may not be a priority [1].

Oral health is an important part of general health for patients with CHD [14], and it is associated with quality of life in general. For instance, it is reported that patients with CHD may have poor oral health, and oral diseases such as dental caries, and severe gingivitis [15]. In addition, soft and defective teeth may be found when the development of cardiac disease coincides with the formation of teeth in children. CHDs might affect ameloblast activity [12], and cause a reduction of enamel deposition [16], as ameloblasts are cells that are sensitive to changes in the intracellular environment [17]. Researchers have reported lower amounts of calcium and phosphate in the compound of enamel and dentin of teeth in children with CHD compared to healthy children [18].

Oral infection may spread to different parts of the body and may cause infective endocarditis, and consequently, patients with CHD may avoid dental and surgical procedures to prevent infective endocarditis [7, 8]. Therefore, it is of critical importance to reduce the risk of infective endocarditis by focusing on dental care, taking steps to improve oral health, and preventing caries and gingival diseases [21].

Moreover, oral health has an impact on the quality of life(QoL) of children with CHD [20]. The presence of untreated teeth leads to a decrease in the QoL of patients due to pain, infection, and swelling [22]. These patients are at higher risk of caries at an early age due to heart medication [23]. Unfortunately, parents of children with CHD usually focus on general health, how to manage it, and reduce their interest in the child’s oral health [20]. This, in turn, can add another burden to the quality of life since dental problems also increase the anxiety and tension of patients, which makes parents feel more guilty about the poor oral health of their child [6, 17].

The World Health Organization (WHO) defines the quality of life(QoL) as people’s perceptions of their role in life according to the community value systems in which they live, their expectations, fears, and goals [24]. A more accurate definition is the absence of disease and symptoms, the comfort in physical functions like chewing and swallowing, the absence of discomfort, pain, or emotional problems associated with the smiling position, and the absence of any social problems associated with oral status [12, 13].

Oral health-related quality of life(OHRQoL) has important implications for clinical practice, dental research, and public health in general [27].

WHO has also recognized this concept as an important part of the global oral health program, so it is important to consider the OHRQoL as a basis for any program aimed at improving oral health [28].

Children with CHD constitute a unique group that requires special medical care with emotional needs [29]. These diseases can affect the quality of life, and cause mood disorders such as fear and anxiety, poor school performance, and poor social interaction with their peers as a result of their health status, especially when they have physical symptoms such as cyanosis [30].

It is important to assess the quality of life of children with CHD to help general dentists to identify the risk group, plan for psychosocial support, and develop strategies that focus on the health needs of these children [31]. Therefore, this study was undertaken to investigate the level of caries, dental plaque, dental abnormalities, gingivitis, and OHRQoL in children with CHD (cases) compared to healthy controls to enable decision-makers to take evidence-based measures for improving the oral health and quality of life of these children.

## Materials and methods

### Study design

The study had a case-control design and it was undertaken in the Pediatric Hospital UPHD in Damascus City in Syria. A total of 200 children with CHDs and 100 controls were included in the study between January 2020 and April 2021.

### Ethics approval

Ethical approval was obtained from the Ethics Committee and the Board of Scientific Research at the Faculty of Dentistry at Damascus University (No. 573) dated 14-5-2019. Official written permissions were also obtained from the authority bodies (Ministry of Health, Ministry of Higher Education and Research). In addition, informed consent was obtained from children’s parents/carers to recruit their children for this study.

### The study sample and sampling method

This study was performed according to the STROBE checklist for observational studies [32]. Congenital heart defects cases were diagnosed by a specialized Cardiologist in the University Pediatric Hospital of Damascus (SS). The sample size was calculated using G*POWER software with a power of 80% and the significant level was set at a *P* value of less than 0.05, also based on a pilot study. The minimum sample size to satisfy the requirement was estimated to be 200 subjects. However, the required sample size was increased to 300 (200 with CHDs, 100 healthy controls) after consulting the cardiologist specialist to avoid Type II sampling error, decrease the effect of confounding variables and increase the precision of the study. The pilot study was undertaken which included 20 children (10 CHD, 10 healthy) who were not part of the main study.

The study included children with CHD aged 4–12 years, who attended the pediatric heart clinic at Pediatric Hospital in Damascus and accepted to take part in the study. Matched healthy children from the same family, of CHD children were also invited, as controls, to take part in this study. The study excluded all children with acquired heart diseases, and children who had hearing or writing disabilities that can prevent them from answering child’s oral health-related quality of life questionnaire (COHRQoL).

All children were assessed by the principal investigator (SSB), who was trained in the Faculty of Dentistry before the clinical examination. An oral visual examination was performed on a chair and in a sitting position located in the hospital using a head-held light, disposable gloves, dental mirror, and probes to investigate caries. Tongue depressors and cotton rolls were also used to remove food residues or to relieve moisture that could prevent the direct vision of the teeth, plaque accumulation, developmental abnormalities, dental caries, and gingivitis, were assessed. Dental caries were evaluated without radiographs to investigate adjacent caries. dmft Index and DMFT Index were recorded based on the criteria of the World Health Organization [33].

The Oral Hygiene Index(OHI) was used for detecting children’s oral hygiene [34]. Gingivitis was assessed through the Papillary-Marginal-Gingivitis-Index (PMGI) [35] using a disposable mirror and tongue depressor without entering the probe into the sulcus. Developmental abnormalities were also recorded, such as beg teeth, microdontia, and enamel defects in both primary and permanent dentition, according to Millett and Welbury [36], and were classified as either loss of the surface of enamel (hypoplasia)or change in enamel translucency (opacity).

The Arabic version of COHRQoL which was translated and validated by a large sample of Arabic children was designed for use with children and teenagers [37].

The questionnaire consists of 36 items that investigate the impact of the condition of teeth, lips, and mouth on the child’s life. It includes 4 domains: Oral Symptoms (6 questions); Functional Limitations (9 questions); Emotional Well-Being (9 questions); and Social Well-Being (12 questions) [37].

Based on previous work [38], children were asked directly, to answer questions related to the Arabic COHRQoL questionnaire without the help of their parents [38].

Moreover, the reliability of the COHRQoL questionnaire was evaluated using Cronbach’s alpha coefficient, and the value for all COHRQoL questionnaires and all domains was above 0.743, showing a good degree of stability (Table 1).

**Table 1:** COHRQoL questionnaire reliability coefficients using crohnbach’s alpha equation

COHRQoL domains	Alpha crohnbach value
**Oral Symptoms domain**	**0.822**
**Functions Limitations domain**	**0.743**
**Emotional Well-Being domain**	**0.795**
**Social Well-Being domain**	**0.758**
**COHRQoL**	**0.853**

COHRQoL - Child Oral Health-Related Quality of Life Questionnairea

The data was processed using IBM SPSS Statistics version 25. Independent *t*-student and Chi‑square tests were performed to determine the presence of significant differences between the two groups. Each question about the COHRQoL domains was scored on a 2-point scale (1 = ‘Yes’, 0 = ‘No’). Then, scores for each domain were calculated by summing the response codes to their questions, *P* ≤ 0.05 was considered statistically significant.

## Results

A total of 200 children aged 4–12 years, with CHDs (112 males, 88 females),who had VSD(36.5%), ASD(19.5%), PS(15%), AOS(10%), or TOF(6.5%), were included. In addition, 100 healthy-matched children (44 males, 56 females) were also involved. The existing differences between CHD children and controls in the mean of DMFT, dmft index, OHI, and PMGI, were investigated. Results are presented in Table 2.

**Table 2:** Descriptive analysis and statistical differences of dental indices between cases(CHD) and controls(healthy)

Variable	Dental index	Children Group	N.	Means	SD	*P*-value
**Differences in dental index**	**dmft index**	cases	200	5.245	3.982	0.000
		controls	100	2.660	2.244	
	**DMFT index**	cases	200	1.615	1.928	0.731
		controls	100	1.540	1.424	
	**OHI index**	cases	200	2.954	1.266	0.000
		controls	100	1.871	0.763	
	**PMGI index**	cases	200	1.689	0.493	0.000
		controls	100	1.170	0.532	

CHD = congenital heart disease; SD = standard deviation, dmft = decay, missing and filling score (primary teeth); DMFT = decay, missing and filling score (permanent teeth)

The findings of this study indicated that the means of (dmft) were significantly higher in the CHD group than in the controls (5.245 vs. 2.660 respectively *P* = 0.000). Also, the means of the OHI were significantly higher in the CHD group than in controls (OHI index = 2.954 vs. 1.871 respectively, *P* = 0.000).

Also, the mean of the PMGI was significantly higher in the CHD group than in the controls (1.689 vs. 1.170 respectively, *P* = 0.000).

In the permanent dentition, no significant differences between the CHD and control group were observed in the mean DMFT (1.615 vs. 1.540, *P* = 0.731). There were no significant differences between the CHD and control groups in the frequency of dental abnormalities including (peg teeth, microdontia, hypoplasia, hypomaturation, and mixed enamel defect). However, significant differences were observed between the CHDs and control groups in the presence of enamel opacities (*P* = 0.039) and hypocalcification (*P* = 0.009). Results are presented in Table 3.

**Table 3:** Frequency of dental abnormalities and statistical differences between cases(CHD) and controls(healthy)

Dental abnormalities		Number			Percent			*P*-Value
		CHD	Controls	Total	CHD	Controls	Total	
**Peg teeth**	no	199	99	298	99.5%	99%	99.3%	0.616
	yes	1	1	2	0.5%	1%	0.7%	
**microdontia**	no	197	100	297	98.5%	100%	99%	0.218
	yes	3	0	3	1.5%	0%	1%	
**enamel opacities**	no	184	98	282	92%	98%	94%	0.039
	yes	16	2	18	8%	2%	6%	
**hypoplasia**	no	197	100	297	98.5%	100%	99%	0.218
	yes	3	0	3	1.5%	0%	1%	
**hypocalcification**	no	179	98	277	89.5%	98%	92.3%	0.009
	yes	21	2	23	10.5%	2%	7.7%	
**hypomaturation**	no	198	100	298	99%	100%	99.3%	0.316
	yes	2	0	2	1%	0%	0.7%	
**Mixed**	no	197	98	295	98.5%	98%	98.3%	0.750
	yes	3	2	5	1.5%	2%	1.7%	

The OHRQoL was also assessed. The findings are presented in Table 3. There were significant differences between children with CHD and controls in the overall 4 OHRQoL domains scores (*P* = 0.000), and in each Oral Symptom (*P* = 0.000), Emotional Well-Being (*P* = 0.000), Social Well-Being (*P* = 0.000), and Functional Limitations domain as well (*P* = 0.000), as shown in Table 4.

**Table 4:** Comparison of the Overall domain of COHRQoL between study grohe overallups

The overall domain of COHRQoL questionnaire		Number			Percent			P-value
		CHD	Controls	Total	CHD	Controls	Total	
**1. Pain in the teeth, lips, jaws, and mouth?**	**No**	28	44	72	14%	44%	24%	0.000
	**Yes**	172	56	228	86%	56%	76%	
**2. Bleeding gums?**	**No**	130	91	221	65%	91%	73.7%	0.000
	**Yes**	70	9	79	35%	9%	26.3%	
**3. Mouth sores?**	**No**	192	99	291	96%	99%	97%	0.151
	**Yes**	8	1	9	4%	1%	3%	
**4. Bad breath?**	**No**	130	72	202	65%	72%	67.3%	0.223
	**Yes**	70	28	98	35%	28%	32.7%	
**5. Food caught between/in the teeth?**	**no**	152	88	240	76%	88%	80%	0.014
	**yes**	48	12	60	24%	12%	20%	
**6. Food stuck to the roof of the mouth?**	**no**	197	100	297	98.5%	100%	99%	0.218
	**yes**	3	0	3	1.5%	0%	1%	
**Oral Symptoms domain**								0.000
**7. Breathing through the mouth?**	**No**	163	90	253	81.5%	90%	84.3%	0.056
	**Yes**	37	10	47	18.5%	10%	15.7%	
**8. Longer to eat?**	**No**	170	93	263	85%	93%	87.7%	0.061
	**Yes**	30	7	37	15%	7%	12.3%	
**9. Trouble sleeping?**	**No**	189	97	286	94.5%	97%	95.3%	0.333
	**Yes**	11	3	14	5.5%	3%	4.7%	
**10. Difficulty chewing firm foods: apple, corn/steak?**	**No**	183	100	283	91.5%	100%	94.3%	0.003
	**Yes**	17	0	17	8.5%	0%	5.7%	
**11. Difficulty to open your mouth wide?**	**no**	187	97	284	93.5%	97%	94.7%	0.203
	**yes**	13	3	16	6.5%	3%	5.3%	
**12. Difficulty to say any words?**	**no**	193	100	293	96.5%	100%	97.7%	0.058
	**yes**	7	0	7	3.5%	0%	2.3%	
**13. Difficulty to eat food you like?**	**No**	167	96	263	83.5%	96%	87.7%	0.002
	**Yes**	33	4	37	16.5%	4%	12.3%	
**14. Difficulty to drink with a straw?**	**No**	184	95	279	92%	95%	93%	0.337
	**Yes**	16	5	21	8%	5%	7%	
**15. Difficulty drinking/eating hot/cold foods?**	**No**	133	92	225	66.5%	92%	75%	0.000
	**yes**	67	8	75	33.5%	8%	25%	
**Functions Limitations domain**								0.000
**16.Irritable/frustrated?**	**No**	80	86	166	40%	86%	55.3%	0.000
	**Yes**	120	14	134	60%	14%	44.7%	
**17. Felt unsure of yourself?**	**No**	102	87	189	51%	87%	63%	0.000
	**Yes**	98	13	111	49%	13%	37%	
**18. Shy/embarrassed?**	**No**	149	100	249	74.5%	100%	83%	0.000
	**Yes**	51	0	51	25.5%	0%	17%	
**19. Concerned with other people think?**	**No**	142	100	242	71%	100%	80.7%	0.000
	**Yes**	58	0	58	29%	0%	19.3%	
**20. Worried that not as good looking as others?**	**no**	175	100	275	87.5%	100%	91.7%	0.000
	**yes**	25	0	25	12.5%	0%	8.3%	
**21. Upset?**	**no**	172	89	261	86%	89%	87%	0.465
	**yes**	28	11	39	14%	11%	13%	
**22. Nervous/ afraid?**	**No**	151	89	240	75.5%	89%	80%	0.006
	**Yes**	49	11	60	24.5%	11%	20%	
**23. Worried less healthy than other people?**	**No**	121	100	221	60.5%	100%	73.7%	0.000
	**Yes**	79	0	79	39.5%	0%	26.3%	
**24. Worried that he/she is different from other people?**	**No**	108	98	206	54%	98%	68.7%	0.000
	**yes**	92	2	94	46%	2%	31.3%	
**Emotional Well-Being domain**								0.000
**25. Missing school?**	**No**	102	100	202	51%	100%	67.3%	0.000
	**Yes**	98	0	98	49%	0%	32.7%	
**26. Had a hard time paying attention in school?**	**No**	186	97	283	93%	97%	94.3%	0.158
	**Yes**	14	3	17	7%	3%	5.7%	
**27. Had difficulty doing your homework?**	**No**	194	98	292	97%	98%	97.3%	0.612
	**Yes**	6	2	8	3%	2%	2.7%	
**28. Not want to speak/read loud in class?**	**No**	177	98	275	88.5%	98%	91.7%	0.005
	**Yes**	23	2	25	11.5%	2%	8.3%	
**29. Avoid taking part in activities like sports, clubs, and so forth?**	**no**	180	96	276	90%	96%	92%	0.071
	**yes**	20	4	24	10%	4%	8%	
**30. Not want to talk to other children?**	**no**	185	95	280	92.5%	95%	93.3%	0.413
	**yes**	15	5	20	7.5%	5%	6.7%	
**31. Avoided smiling/laughing when around other children?**	**No**	180	99	279	90%	99%	93%	0.004
	**Yes**	20	1	21	10%	1%	7%	
**32. Not want to spend time with other children?**	**No**	186	100	286	93%	100%	95.3%	0.007
	**Yes**	14	0	14	7%	0%	4.7%	
**33. Argued with other children or your family?**	**No**	184	91	275	92%	91%	91.7%	0.768
	**Yes**	16	9	25	8%	9%	8.3%	
**34.Teased/called names by other children?**	**No**	192	91	283	96%	91%	94.3%	0.077
	**Yes**	8	9	17	4%	9%	5.7%	
**35. Left out by other children?**	**No**	165	100	265	82.5%	100%	88.3%	0.000
	**Yes**	35	0	35	17.5%	0%	11.7%	
**36. Asked questions by other children about teeth?**	**No**	98	100	198	49%	100%	66%	0.000
	**yes**	102	0	102	51%	0%	44%	
**Social Well-Being domain**								0.000
**Overall COHRQoL**								0.000

Regarding the assessment of Oral Symptoms domain items, there were statistical differences between children with CHD and controls in q1, 2, and 5.

Regarding the functional limitations domain, there were significant differences between children with CHD and controls in 3 questions (q 10, q13, and q15).

The investigation of emotional well-being domain items indicated the presence of significant differences between children with CHD and controls in 8 questions (q16, 17, 18,19, 20, 22, 23, and 24).

In addition, the investigation of the items related to the social well-being domain demonstrated the presence of significant differences between children with CHD and controls in 6 questions (q 25, 28, 31, 32, 35, and 36). The results of the overall domains are presented in Table 4.

## Discussion

With improved investigation methods, and the advancement of surgical techniques and procedures, the number of surviving children with CHD, and abnormalities in Syria are increasing.

The University Pediatric Hospital of Damascus UPHD was selected as a location for this study as it is the largest hospital in the country.

In this study, the sample of children with CHDs and controls was sufficient to provide a further understanding of oral health and OHRQoL of children with CHD when compared with healthy controls.

Children with CHD need optimal oral health care to prevent consequences and complications which can cause a constant threat to their lives [39]. For instance, caries in primary dentition may increase the risk of future caries in permanent dentition. Plaque and gingivitis during childhood may also lead to periodontitis and loss of teeth later in life [10, 24].

There are at least three reasons for the poor oral health of children with congenital heart diseases. Firstly, parents of children with CHDs usually are not so aware of the critical importance of oral hygiene to prevent dental diseases that can affect the general health and quality of life of their child [20]. Second, general dentists are reluctant to provide children with CHDs who are already at high risk of bacteremia and endocarditis [41]. Third, pediatric cardiac disease specialists do not provide adequate information to families of children with CHD about the importance of oral care. Therefore, both pediatric dentists and pediatric cardiologists should collaborate and have a great role in raising awareness, educating, and improving the general and oral health of patients with CHD [23, 24].

The findings of this study indicated significantly higher dental caries among CHDs children compared to controls. A previous study has also reported a significant increase in dental caries among Brazilian children with CHDs, especially those who had cyanotic defects, cardiac surgery, low socioeconomic conditions, and low educational levels with their fathers [43].

Similarly, researchers in Iran, who also investigated 74 healthy and 68 children with CHD, found the value of DMFT was significantly higher (6.4) in CHD children when compared to (5.5) in healthy children [44].

Dental caries was significantly frequent in children with CHDs, in a study undertaken by Sivertsen and colleagues (2016) from Western Norway (25.4 versus 18.3% in controls). They have also reported an increased frequency of dental erosion and enamel defects in the same children [45].

Also, Stecksén-Blicks and colleagues (2004) found that children with congenital heart disease had more dental caries in their permanent teeth than age-and sex-matched healthy children. They suggested that this difference might be due to taking some heart medication like digoxin frequently, a sucrose-containing syrup [39].

However, Franco (1996) and Tasioula and colleagues (2008) have not found any significant difference between healthy and children with CHD in the prevalence of dental caries and enamel defects in both primary and permanent dentition [39, 40].

In the present study, poor oral health and gingivitis were found to be highly prevalent among children with CHD compared to healthy peers due to a lack of awareness about the importance of brushing teeth and oral health care in general.

Similarly, Ali and colleagues(2017) found a significant increase in the number of sites with plaque, gingivitis, and the mean dmft/DMFT in children with CHD when compared to controls [18].

Hayes and Fasules (2001) also indicated increased gingivitis in children who had cardiac surgery [48].

Enamel has a protective role in the tooth. Loss of enamel exposes the sensitive dentin underneath. Once damaged, the enamel is generally unable to recover. Systemic diseases may cause damage to tooth enamel or dentin due to their nature and severity [17].

In our study, significant differences were found between CHD children and healthy controls in the frequency of both enamel opacities and hypocalcification.

These results were in agreement with the study of AL-Etbi and Al-Alousi (2011) who reported a high frequency of enamel defects such as hypoplasia of primary teeth in children with ventricular septal defect (acyanotic CHD) [49]. However, these results were different from a study undertaken by Tasioula and colleagues (2008) and Cantekin and colleagues (2015), who reported no significant differences between CHD children with CHDs and controls in the frequency of enamel defects [36, 40].

In the present study, the COHRQoL was utilized as a reliable and valid tool in Arabic-speaking countries such as Syria, which has been proven by Brown and Al-Khayal in Saudi Arabia(2006) [37].

The investigation of all domains related to OHRQoL indicated that children with CHD had significantly reduced oral health-related quality of life when compared to controls. These findings were similar to those reported by Mellion and colleagues (2014) as well as Guerra and colleagues (2013) who reported lower scores of quality of life in children with CHD [51], low HRQoL in CHD children who had surgery early infancy [52].

In contrast, the findings of this study were different from others reported by Uzark and colleagues (2008), who reported no significant differences in overall QoL in children with cardiovascular diseases [53]. Similarly, Larsen and colleagues (2010), who investigated 239 children, found similar functional health between CHD and controls of the same age [54].

Interestingly, Areias and colleagues (2013) have shown a better quality of life among CHD patients than controls in Portugal, as their results demonstrated better QoL in CHD patients compared to controls in the Social Relationships, Physical, Environmental, and General Dimensions [55].

The eexisting differences between the findings may be attributed to the differences in the methodology applied in the investigations or to the specific preventive measures and strategies that are implemented to support children with CHD.

Several previous studies have investigated the health-related quality of life in patients with CHDs. However, there is no available recent study in the last 10 years in general, and in Syria especially that investigated the oral health-related quality of life in children with CHDs.

In the present study, the overall scores of the “Oral Symptoms” domain showed significant differences between the two groups of children, such as gingival bleeding, pain in the mouth, and food caught in the teeth.

Our study reported an increased frequency of gingivitis in children with CHD due to poor oral hygiene.

A similar study indicated that children with CHD patients who are transplant recipients had difficulty in maintaining oral care due to gingival hypertrophy [56].

The findings of our study that demonstrated the increased frequency of dental caries in children with CHD may explain the significant differences observed in answering the question related to food held between teeth and also the question about pain in the teeth, lips, jaws, and mouth.

European countries which have preventive programs and the availability of dental services also had a higher rate of caries in children with CHDs [7, 30], thus adding another burden to their quality of life.

Our study is in agreement with the study of Sheller and colleagues (1997) and Larsen and colleagues (2010) which reported that the sequels of untreated dental caries like infection, swelling, and pain may decrease the quality of life [15, 49].

In addition, findings related to the “Function Limitations” domain indicated the presence of a significant difference between children with CHDs and controls in several questions related to difficulty in chewing, drinking, eating desired foods, and eating hot or cold foods. This can be explained by dental anomalies and caries, which were more common in CHD patients.

Regarding the “Emotional well-being” domain, the findings revealed that children with CHD were significantly more irritable, frustrated, shy, embarrassed, more concerned about other people thinking, nervous, afraid, and worried that they are different from others.

All these findings are consistent with Utens and colleagues (1998) who demonstrated the presence of behavioral and emotional problems among CHD children [58].

da Fonseca and colleagues (2009) study showed that children with CHD had a reduced oral health-related quality of life in some specific domains, including family and psychological stress on the child [20].

In contrast, Moons and Norekvål(2006) reported that CHD patients could face challenges and stresses and understand their illness, especially after having survived surgical interventions. Also, these patients have a stronger sense of coherence than healthy people [59].

It might be true that children with CHD who were born with cardiac disease have learned to be more coherent and adaptable in life [60].

Ong and colleagues (2017) reported that the emotional and behavioral problems are just in early childhood but later these have been overlooked when families make early diagnoses and treatment, to become a cause for concern later when the child begins integration into the school and community [61].

In addition, the “Social Well-Being” domain indicated the significant differences between children with CHDs and controls in some questions related to social activities such as smiling/laughing around other children, speaking /reading loud in class, losing school lessons, avoiding other children, and receiving more questions about teeth.

Similar to our findings, Svensson and colleagues (2017) reported that children with univentricular hearts were unwilling to go to school to avoid embarrassing situations and questions [62].

Similarly, Diller and colleagues (2013), showed that exercise intolerance can result in low socioeconomic status among CHD patients [63].

Eslami and colleagues (2014) attributed the avoidance behavior of a child with CHD to the reduction of parental education, which leads, in turn, to the lack of attention to child education by his/her parents [64].

Moreover, Amedro and colleagues (2015) indicated that physical well-being, self-reliance, financial resources, social support, school performance, self-reported psychological aspects, emotions, and moods are compromised in children with CHDs [65].

One of the limitations of this study is related to the selection of children who attended the UPHD, which is the largest public hospital in Syria. We may have excluded CHD children who seek health care in a private hospital. This may result in other different findings. Future work should consider other children with CHDs who attend private clinics and hospitals.

The study excluded all children with acquired heart diseases, and children who had hearing or writing disabilities that can prevent them from answering child’s oral health-related quality of life questionnaire (COHRQoL). This might be a limitation as it can influence the results. Future work should consider investigating other acquired heart diseases with associated disabilities. On the other hand, the study has its strengths as it has a sufficient sample size with matched controls. However, our study did not take into consideration the severity of CHDs when recruiting patients. This might be a limitation, so future work should study the effect of disease severity on oral manifestations of the patients and their OHRQoL.

In addition, OHRQOL has not been previously assessed in Syria among children with CHD. Our findings have provided the initial basis for developing future oral health programs for children with CHDs. The mutual relationship between oral and systemic health should be appropriately understood by all health professionals and parents to improve the quality of life of these patients and promote their general and psychological health.

## Conclusion

Children with CHD have poor oral health, with high percentages of plaque accumulation, untreated carious lesions, gingivitis, and dental abnormalities, in their primary and permanent dentition. Raising awareness about oral health problems in children with CHD is of critical importance to prevent potential emotional and social, and general complications.

This study provides baseline information necessary to implement preventive programs within the health system and to provide the best special oral healthcare, and special psychological and social support for children with CHD in Damascus, Syria.

In addition, future studies that include children with acquired heart diseases should be considered. Future work is still required to investigate other Syrian hospitals and take measures at the national level to improve the health of these vulnerable children.

## Data Availability

The datasets used and/or analyzed during the current study are available. from the corresponding author on reasonable request.

## Abbreviations

CHD: Congenital Heart Disease
QoL: Quality of Life
WHO: World Health Organization
OHRQoL: Oral health-related quality of life
COHRQoL: Child Oral health-related quality of life questionnaire
VSD: Ventricular Septal Defect
ASD: Atrial Septal Defect
TOF: Tetralogy of Fallot
PS: Pulmonary Stenosis
AOS: Aortic Stenosis
dmft: Decayed, missing, and filled temporary teeth
DMFT: Decayed, missing, and filled permanent teeth
PMGI: Papillary-Marginal-Gingivitis-Index
OHI: Oral-Hygiene-Index
UPHD: University Pediatric Hospital of Damascus
MD: Mayssoon Dashash
SSB: Shouq Sharar Bsesa
SS: Samir Srour
SD: Standard deviation
